# Bacterial Outer Membrane Vesicle-Mediated Cytosolic Delivery of Flagellin Triggers Host NLRC4 Canonical Inflammasome Signaling

**DOI:** 10.3389/fimmu.2020.581165

**Published:** 2020-11-18

**Authors:** Jungmin Yang, Inhwa Hwang, Eunju Lee, Sung Jae Shin, Eun-Jin Lee, Joon Haeng Rhee, Je-Wook Yu

**Affiliations:** ^1^ Department of Microbiology and Immunology, Institute for Immunology and Immunological Diseases, Brain Korea 21 PLUS Project for Medical Science, Yonsei University College of Medicine, Seoul, South Korea; ^2^ Department of Life Sciences, Korea University, Seoul, South Korea; ^3^ Department of Microbiology, Clinical Vaccine R&D Center, Chonnam National University Medical School, Gwangju, South Korea

**Keywords:** outer membrane vesicles, NLRC4, inflammasome, interleukin-1, caspase-1, flagellin, host defense

## Abstract

Bacteria-released components can modulate host innate immune response in the absence of direct host cell–bacteria interaction. In particular, bacteria-derived outer membrane vesicles (OMVs) were recently shown to activate host caspase-11-mediated non-canonical inflammasome pathway *via* deliverance of OMV-bound lipopolysaccharide. However, further precise understanding of innate immune-modulation by bacterial OMVs remains elusive. Here, we present evidence that flagellated bacteria-released OMVs can trigger NLRC4 canonical inflammasome activation *via* flagellin delivery to the cytoplasm of host cells. *Salmonella typhimurium*-derived OMVs caused a robust NLRC4-mediated caspase-1 activation and interleukin-1β secretion in macrophages in an endocytosis-dependent, but guanylate-binding protein-independent manner. Notably, OMV-associated flagellin is crucial for *Salmonella* OMV-induced inflammasome response. Flagellated *Pseudomonas aeruginosa*-released OMVs consistently promoted robust NLRC4 inflammasome activation, while non-flagellated *Escherichia coli*-released OMVs induced NLRC4-independent non-canonical inflammasome activation leading to NLRP3-mediated interleukin-1β secretion. Flagellin-deficient *Salmonella* OMVs caused a weak interleukin-1β production in a NLRP3-dependent manner. These findings indicate that *Salmonella* OMV triggers NLRC4 inflammasome activation *via* OMV-associated flagellin in addition to a mild induction of non-canonical inflammasome signaling *via* OMV-bound lipopolysaccharide. Intriguingly, flagellated *Salmonella*-derived OMVs induced more rapid inflammasome response than flagellin-deficient *Salmonella* OMV and non-flagellated *Escherichia coli*-derived OMVs. Supporting these *in vitro* results, *Nlrc4*-deficient mice showed significantly reduced interleukin-1β production after intraperitoneal challenge with *Salmonella*-released OMVs. Taken together, our results here propose that NLRC4 inflammasome machinery is a rapid sensor of bacterial OMV-bound flagellin as a host defense mechanism against bacterial pathogen infection.

## Introduction

Host immune system attempts to detect invasion of bacterial pathogens *via* pattern-recognition receptors on tissue-resident sentinel cells, such as macrophages (1). Recognition of bacterial ligand by these receptors promptly results in the production of proinflammatory cytokines, which then trigger host inflammatory responses by recruiting more leukocytes from the bloodstream to clear the invading pathogens (2). Among the proinflammatory cytokines produced during the initial stage of bacterial infection, interleukin (IL)-1β has a crucial role in initiation of the inflammatory process (3). The maturation and secretion of IL-1β requires formation of inflammasome multi-protein complex (4). Upon bacterial infection, bacterial ligands drive inflammasome complex assembly. This complex consists mostly of sensor protein, adaptor protein, and procaspase-1, leading to the generation of active caspase-1 (5). Caspase-1 then processes inactive pro-IL-1β into an active form and facilitates leaderless IL-1β secretion into the extracellular space.

Several cytosolic inflammasome sensor molecules detect intracellular bacteria-specific ligands in direct or indirect ways (2, 5). NOD-like receptor family, CARD domain-containing 4 (NLRC4) is the most professional sensor molecule that responds to bacterial infections (6). NLRC4 monomer is assembled into active oligomeric complex upon engagement by bacterial flagellin-bound neuronal apoptosis inhibitory protein 5 (NAIP5), which is the actual sensor for bacterial flagellin (7). Similarly, both NAIP1 and NAIP2 recognize type 3 secretion system (T3SS) needle and rod proteins in Gram-negative bacteria (8, 9), and this NAIPs–T3SS protein complex promotes the assembly of NLRC4 inflammasome. *Nlrc4*-deficient mice showed increased susceptibility to infection with bacterial pathogens such as *Salmonella typhimurium* (10, 11), which indicates that NLRC4 inflammasome confers innate immune protection against pathogenic bacteria.

In addition to NLRC4, absent in melanoma 2 (AIM2) directly detects cytosolic bacterial double-stranded DNA to form AIM2 inflammasome (12). Caspase-11 recognizes intracellular lipopolysaccharide (LPS), derived from invasive Gram-negative bacteria, leading to the activation of non-canonical inflammasome pathways (13, 14). In addition to this direct sensing of bacterial ligands by inflammasome sensor molecules, some bacterial toxins can indirectly drive inflammasome activation. For example, pore-forming toxins from *Staphylococcus aureus* or *Streptococcus pneumoniae* induce NLR family, pyrin domain-containing 3 (NLRP3)-mediated inflammasome activation (5). But some bacterial toxins promote pyrin inflammasome signaling *via* Rho GTPase inhibition (15).

Outer membrane vesicles (OMVs) are produced mostly from Gram-negative bacteria by vesiculation process (16). The physiological function of bacterial OMVs remains to be further determined, but the generated OMVs eliminate toxic compounds (***e.g***., misfolded proteins) to promote bacterial survival under stress conditions (16). In addition, OMVs can increase bacterial pathogenicity by delivering antibiotic resistance genes to other bacteria and virulence factors to host cells (17). Hence, host immune recognition of OMVs is beneficial for detection of bacterial invasion and inhibition of the spread of bacterial pathogenicity. Intriguingly, recent studies revealed that bacterial OMVs can induce caspase-11-mediated non-canonical inflammasome activation *via* deliverance of OMV-bound LPS into the cytosol (18, 19). In this study, we present evidence that OMVs from flagellated bacteria are able to trigger NLRC4-mediated canonical inflammasome activation in a mainly flagellin-dependent manner. This bacterial OMV-driven NLRC4 inflammasome signaling may provide an efficient host defense mechanism as a rapid sensing machinery against bacterial infection.

## Materials and Methods

### Mice

C57BL/6 and *Nlrp3* ^−/−^ mice were obtained from The Jackson Laboratory. *Nlrc4* ^−/−^ mice were obtained from Genentech through Dr. Tatsuya Saitoh (Osaka University). *Gbp2* ^−/−^ mice were provided by Korea Mouse Phenotyping Center. All mice were bred at Yonsei University College of Medicine and maintained under specific pathogen-free conditions. 9- to 12-week old mice were used for the experiments. For some experiments, mice were injected with *Salmonella* OMVs (50 µg) or phosphate-buffered saline (PBS) *via* the intraperitoneal route. Protocols for the animal experiments were approved by the Institutional Ethical Committee, Yonsei University College of Medicine. All experiments were performed in accordance with the approved guidelines of the Institutional Ethical Committee.

### Reagents and Antibodies

LPS, ATP, nigericin, DOTAP liposomal transfection reagent, proteinase K, cytochalasin D, MCC950, and OptiPrep density gradient were purchased from Sigma-Aldrich. Pitstop2 was obtained from Abcam. Flagellin purified from *S. typhimurium* CDC 6516-60 (ATCC 14028) was purchased from InvivoGen. CytoTox96 non-radioactive cytotoxicity assay kit was obtained from Promega. The following antibodies were used for detecting caspase-1 (Adipogen), NLRP3 (Adipogen), IL-1β (R&D Systems), ASC (Santa Cruz Biotechnology), flagellin (InvivoGen), OmpA (Biorbyt), and gasdermin D (Novus Biologicals). Anti-NLRC4 antibody was prepared using mouse NLRC4 peptide (AbFrontier).

### Cell Culture

Mouse bone marrow-derived macrophages (BMDMs) were prepared from mouse femurs as previously described (12). Immortalized *Asc* ^−/−^ BMDMs were provided by Dr. E.S. Alnemri (Thomas Jefferson University, Philadelphia, USA). All BMDMs were maintained in L929-conditioned DMEM supplemented with 10% fetal bovine serum and antibiotics. Cell death was determined by extracellular release of lactate dehydrogenase (LDH) using a CytoTox96 non-radioactive cytotoxicity assay kit. The LDH release was calculated as [extracellular LDH/(intracellular LDH + extracellular LDH) × 100].

### Bacterial Cultures

*Salmonella enterica* serovar typhimurium strains, SL1344 and 14028s, were used in this study. Flagellin- or prgJ deletion *Salmonella* mutants (*fliC–fljB*, *prgJ*, and *fliC–fljB–prgJ*) were generated by homologous recombination and kindly provided by Dr. F. Shao (National Institute of Biological Sciences, Beijing, China). *Pseudomonas aeruginosa* strain PAO1 was kindly provided by Dr. S.S. Yoon (Yonsei University College of Medicine). *Escherichia coli* B (BL21) and DH5*α* strains were obtained from RBC bioscience. Bacterial strains were grown overnight in LB medium with shaking and aeration. Overnight-cultured bacteria were normalized by bacterial number, diluted 1:50 with fresh LB medium and grown for an additional 6 or 1–12 h. Then, the bacterial suspension was centrifuged to pellet the bacteria and further filtered using 0.2-µm syringe filter to remove bacteria. The final filtrate was used for bacteria-free CS. The prepared bacterial CS was added onto LB agar plate and incubated overnight to confirm the absence of bacterial contamination. To inactivate protein moiety in the bacterial CS, CSs were heated at 98°C for 30 min (heat treatment) or treated with proteinase K (10 µg/ml) at 37°C for 30 min and subsequently boiled at 98°C for 30 min to eliminate proteinase K activity.

### Preparation of Bacterial Outer Membrane Vesicles

To prepare outer membrane vesicles, bacteria-free CSs (1L) were concentrated to 25–50 ml by using the protein concentrator PES 100K MWCO (Thermo Fisher Sci) and ultracentrifuged at 150,000 × g at 4°C for 3 h. The pelleted fraction was washed with PBS and re-ultracentrifuged. The final pellet was resuspended with PBS (1 ml). The concentration of isolated OMVs was determined using a BCA protein assay kit. Isolated OMVs were analyzed using a Nanoparticle Tracking Analysis system (NS300, Malvern Panalytical Ltd). The analyses were performed with purified OMVs from at least three-independent isolation. To further fractionate OMVs, OptiPrep gradients were layered as: 1.7 ml 45%, 1.7 ml 40%, 1.7 ml 35%, 1.7 ml 30%, 1.7 ml 20%, and 1.7 ml 10%. Isolated OMVs (500 μl) were loaded on top of the OptiPrep gradients. The gradients were ultracentrifuged at 150,000 × *g* for 20 h at 4°C, and 1 ml of each fraction was obtained. Obtained fractions were precipitated for immunoblotting by methanol/chloroform extraction method. In several experiments, isolated OMVs were heated at 98°C for 30 min (heat treatment) or treated with proteinase K (10 µg/ml) at 37°C for 30 min and subsequently added with PMSF (1 mM) to eliminate proteinase K activity.

### Immunoblot Analysis

Cells were lysed in buffer containing 20 mM HEPES (pH 7.5), 0.5% Nonidet P-40, 50 mM KCl, 150 mM NaCl, 1.5 mM MgCl_2_, 1 mM EGTA, and protease inhibitors. Soluble lysates were fractionated by SDS-PAGE and then transferred to PVDF membranes. In some experiments, cell culture supernatants were precipitated by methanol/chloroform as described previously (20) and then immunoblotted. All the blots shown are representative image of at least three-independent experiments.

### Assay of Inflammasome Activation

To induce a conventional NLRP3 inflammasome activation, BMDMs were primed with LPS (0.25 µg/ml, 3 h), followed with ATP treatment (2–2.5 mM, 30–45 min) (21). To induce a non-canonical inflammasome activation, cells were treated with Pam3CSK4 (1 µg/ml, 4 h), followed by the transfection of LPS (2 µg/ml, 5 h) using a DOTAP (DT). Inflammasome activation was determined by the presence of active caspase-1 p20 and active IL-1β from culture supernatants in immunoblots and by the extracellular IL-1β quantification using ELISA. To measure extracellular levels of IL-1β or IL-6, culture supernatants were assayed using an IL-1β- or IL-6-specific ELISA kit (BioLegend) according to the manufacturer’s instructions.

### Confocal Microscopy

Isolated OMVs were labeled by incubating with 1% (v/v) Vybrant Dil (Thermo Fisher Scientific) at 37°C for 30 min. Unbound dyes were removed by ultracentrifugation and PBS washing three times. PBS was also incubated with dye in the same way as the OMVs and used for negative control. After treating with labeled OMVs, cells were fixed with 4% para-formaldehyde. Fixed cells on coverslip were mounted using ProLong Gold reagent with DAPI. The cells were then examined using confocal microscopy (LSM700, Carl Zeiss, Oberkochen, Germany).

### Statistical Analysis

All values were expressed as the mean ± SEM of individual samples. All the data were obtained from at least three-independent experiments. Data were analyzed using Student’s *t*-test or one-way analysis of variance (ANOVA) followed by Dunnett’s *post hoc* test for comparison of all groups with control group as indicated in the figure legend. The level of statistical significance was set at *p* ≤ 0.05. Analyses were performed using GraphPad Prism 5.

## Results

### Culture Supernatant From *Salmonella typhimurium* Promotes NLRC4-Dependent Caspase-1 Activation

**Figure 1:**
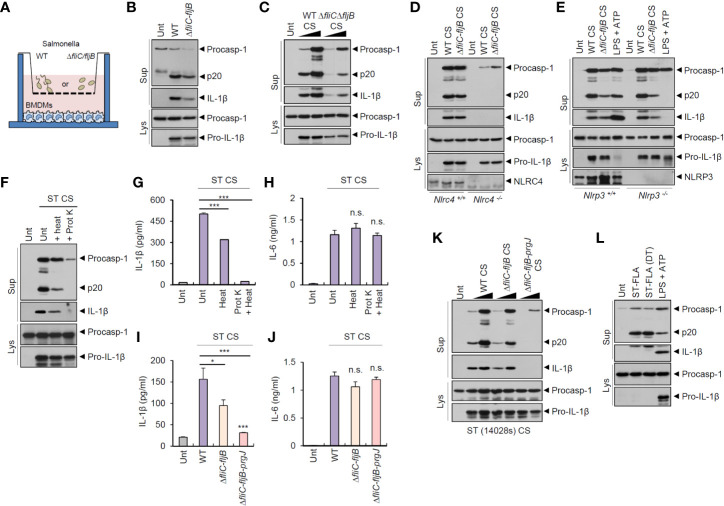
Culture supernatants from *Salmonella typhimurium* promotes NLRC4-dependent inflammasome activation. **(A)** Illustration of experimental scheme to determine inflammasome activation in BMDMs by bacteria-secreted molecules using transwell plate. **(B)** Immunoblots from mouse BMDMs in the lower well **(A)** incubated with wild-type (WT) or *ΔfliC-fljB S. typhimurium* SL1344 in the upper well (**A**, MOI 30) for 7 h. **(C)** Immunoblots from mouse BMDMs untreated or treated with culture supernatant (CS, 1/33 or 1/10 volume of culture medium), derived from 6 h culture of WT or *ΔfliC–fljB* *S. typhimurium*, for 6 h. **(D)** Immunoblots from *Nlrc4* ^+/+^ or *Nlrc4* ^−/−^ mice BMDMs treated with WT or *ΔfliC*–*fljB S. typhimurium* CS (1/10) for 6 h. **(E)** Immunoblots from *Nlrp3* ^+/+^ or *Nlrp3* ^−/−^ mice BMDMs treated with WT or *ΔfliC*–*fljB S. typhimurium* CS (1/10) for 6 h, or primed with LPS (0.25 µg/ml, 3 h), followed by treatment with ATP (2.5 mM, 40 min). **(F)** Immunoblots from mouse BMDMs incubated with *S. typhimurium* CS (1/20) with or without heat treatment (97°C, 30 min) or proteinase K treatment (10 µg/ml, 30 min), for 6 h. **(G, H)** Quantification of IL-1β **(G)** or IL-6 **(H)** in the supernatant of mouse BMDMs treated with *S. typhimurium* CS (1/20) as same as in **(F)**. (*n* = 3, one-way ANOVA) **(I, J)** Quantification of IL-1β **(I)** or IL-6 **(J)** in the culture supernatants of mouse BMDMs treated with WT, *ΔfliC*–*fljB*, or *ΔfliC*–*fljB*–*prgJ S. typhimurium* CS (1/100) for 6 h. (*n* = 4, one-way ANOVA). **(K)** Immunoblots from mouse BMDMs treated with culture supernatant (CS, 1/100 or 1/20), derived from 6 h culture of WT, *ΔfliC*–*fljB*, *ΔfliC*–*fljB*–*prgJ* *S. typhimurium* 14028s, for 6 h. **(L)** Immunoblots from mouse BMDMs treated with *S. typhimurium* flagellin (ST-FLA, 250 ng/ml) with or without premixing with DOTAP (DT) liposomal transfection reagent for 6 h or treated with LPS (0.25 µg/ml, 3 h), followed by ATP treatment (2.5 mM, 40 min). Culture supernatants (Sup) or cellular lysates (Lys) were immunoblotted with the indicated antibodies. Data were expressed as the mean ± SEM. Asterisks indicate significant differences compared with *S. typhimurium* CS-treated group. (*P < 0.05, ***P < 0.001, n.s. not significant).

*Salmonella typhimurium* is the most common enteric pathogen causing gastroenteritis. To examine whether *S. typhimurium*-secreted molecules can trigger host inflammasome signaling pathways regardless of bacterial contact with host macrophages, we used a transwell culture system (**Figure 1A**). As flagellin is the major ligand for inflammasome activation during *S. typhimurium* infection (22, 23), we tested flagellin-deficient (Δ*fliC–fljB*) *S. typhimurium* as well as wild-type bacteria. Culture media from wild-type and flagellin-deficient (Δ*fliC–fljB*) *S. typhimurium* in the upper wells clearly drove a robust inflammasome activation of mouse bone marrow-derived macrophages (BMDMs) in the lower wells, as determined by the presence of cleaved caspase-1 (p20) and matured IL-1β in the supernatant (**Figure 1B**). To further confirm the inflammasome-stimulating capacity of *S. typhimurium*-secreted molecules, we carefully collected bacteria-free culture supernatants (CS) from *S. typhimurium* and treated them into BMDMs. Notably, CS from wild-type or flagellin mutant *S. typhimurium* caused a marked caspase-1 activation (**Figure 1C**). This *Salmonella* CS-induced inflammasome activation was dependent on NLRC4 and apoptosis-associated speck-like protein containing a caspase recruitment domain (ASC), which is a crucial adaptor protein of the inflammasome complex (**Figure 1D** and **Figure S1A**). However, activation was not dependent on NLRP3 (**Figure 1E**). These findings indicated that *Salmonella*-secreted components are able to promote NLRC4-mediated inflammasome activation in the absence of direct bacterial contact with host macrophages.

### Culture Supernatant From *Salmonella typhimurium* Activates Inflammasome Signaling in a Flagellin- and prgJ-Dependent Manner

To determine the bacterial components responsible for *Salmonella* CS-induced NLRC4 inflammasome activation, we first examined a potential role of protein present in the CS. Inactivation of protein by heat treatment applied to *S. typhimurium* CS significantly reduced caspase-1 activation and active IL-1β secretion (**Figures 1F, G**). Additionally, protein degradation *via* proteinase treatment completely abrogated *S. typhimurium* CS-induced inflammasome activation (**Figures 1F, G**). Indeed, proteinase treatment, but not heat treatment, caused a complete degradation of bacterial proteins in the CS and lysates (**Figures S1B, C**). However, neither heat inactivation nor proteinase treatment impaired IL-6 production by *Salmonella* CS (**Figure 1H**).

Flagellin and T3SS protein are the main stimulants for NAIP-dependent NLRC4 inflammasome activation during *S. typhimurium* infection (22, 23). We thus examined a putative role of flagellin or PrgJ, a T3SS rod protein, in *S. typhimurium* CS-induced inflammasome activation. CS from flagellin-deficient (Δ*fliC–fljB*) *Salmonella* caused significantly less IL-1β production than wild-type *Salmonella* CS (**Figure 1I**). Furthermore, *S. typhimurium* CS from flagellin/prgJ-deficient mutants (Δ*fliC–fljB–prgJ*) induced remarkably attenuated IL-1β secretion from BMDMs (**Figure 1I**). However, both proteins were not required for CS-induced IL-6 production from BMDMs (**Figure 1J**). Consistent with these findings, flagellin/prgJ-deficient *Salmonella*-derived CS failed to induce a robust activation of caspase-1 and cell death in BMDMs (**Figure 1K** and **Figure S2**).

To further confirm the role of flagellin or prgJ in the CS, we directly treated a purified *Salmonella* flagellin protein into BMDMs with or without DOTAP (N-[1-(2,3-Dioleoyloxy)propyl]-N,N,N-trimethylammonium methyl-sulfate) liposomal transfection agent. Consistent with the results of previous study (22), cytosolic delivery of flagellin using DOTAP caused caspase-1 activation in BMDMs (**Figure 1L**). Notably, 6 h treatment with *S. typhimurium* flagellin alone also drove caspase-1 processing (**Figure 1L**). This unexpected inflammasome activation by recombinant flagellin required the expression of NLRC4 and ASC but not of NLRP3 (**Figures S3A–S3C**). This result suggested that *Salmonella*-released flagellin and T3SS rod protein PrgJ are capable of inducing host NLRC4 inflammasome activation.

### *Salmonella typhimurium*-Released Outer Membrane Vesicles Promote Flagellin-Mediated Caspase-1 Activation and IL-1β Secretion in an Endocytosis-Dependent Manner

**Figure 2:**
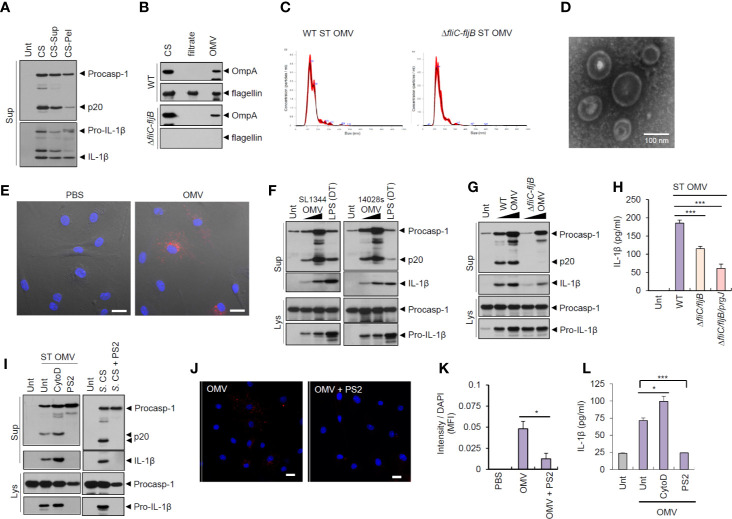
Outer membrane vesicles from *Salmonella typhimurium* activate inflammasome signaling *via* receptor-mediated endocytosis. **(A)** Immunoblots from the culture supernatants of mouse BMDMs treated with *S. typhimurium* CS, or supernatant (CS-Sup) or pellet (CS-Pel) from ultracentrifugation (150,000 × g, 18 h) of *S. typhimurium* CS. **(B)** Immunoblots from wild-type (WT) or *ΔfliC*–*fljB S. typhimurium* CS, filtrates from Centricon concentration (**Supplementary Figure S3A**) and the extract of OMVs. **(C)** Nanoparticle Tracking Analysis of OMVs isolated from WT or *ΔfliC*–*fljB S. typhimurium*. **(D)** Transmission electron microscopy of OMVs isolated from wild-type *S. typhimurium*. Scale bar, 100 nm. **(E)** Representative confocal microscopy images of BMDMs treated with Vybrant Dil-labeled PBS or *S. typhimurium* OMVs. Scale bar, 20 µm. **(F)** Immunoblots from mouse BMDMs treated with OMVs isolated from *S. typhimurium* SL1344 or 14028s (0.5 or 5 µg/ml) for 8 h or treated with Pam3CSK4 (1 µg/ml, 4 h), followed by the transfection of LPS (2 µg/ml, 5 h) using a DOTAP (DT). **(G)** Immunoblots from mouse BMDMs treated with WT or *ΔfliC*–*fljB S. typhimurium* (SL1344) OMV (1 or 5 µg/ml, 8 h). **(H)** Quantification of IL-1β in the culture supernatants of mouse BMDMs treated with WT, *ΔfliC*–*fljB*, or *ΔfliC*–*fljB*–*prgJ S. typhimurium* (SL1344) OMVs (5 µg/ml, 8 h) (*n* = 7, one-way ANOVA). **(I)** Immunoblots from mouse BMDMs treated with *S. typhimurium* OMV (5 µg/ml, 6 h) in the presence of cytochalasin D (5 µM) or Pitstop 2 (PS2, 10 µM), or treated with *S. typhimurium* CS (1/20) in the presence of PS2 (10 µM) pretreatment (10 min before *Salmonella* CS treatment). **(J)** Representative confocal microscopy images of BMDMs treated with Vybrant Dil-labeled *S. typhimurium* OMVs (5 µg/ml, 6 h) in the presence of Pitstop 2 treatment (10 µM, 30 min pretreat). Scale bar, 20 µm. **(K)** Relative Dil fluorescence intensity per DAPI signals of BMDMs as treated in **(J)**. (*n* = 6, one-way ANOVA). **(L)** Quantification of IL-1β in the culture supernatants of mouse BMDMs treated as in (I, left panel). (*n* = 3, one-way ANOVA). Culture supernatants (Sup) or cellular lysates (Lys) were immunoblotted with the indicated antibodies. Data were expressed as the mean ± SEM. Asterisks indicate significant differences compared with *S. typhimurium* CS-treated group. (*P < 0.05, ***P < 0.001).

To examine whether bacteria-secreted vesicle fractions mediated inflammasome activation, we isolated pelleted fractions after 150,000 × *g* centrifugation of *Salmonella* CS. Intriguingly, pelleted-vesicles from *S. typhimurium* CS still induced caspase-1 activation and active IL-1β secretion (**Figure 2A**). We then further isolated bacterial OMVs from *Salmonella* CS (**Figure S4A**). Outer membrane protein A (OmpA) was present in *Salmonella* OMV fractions (**Figure 2B**). The results of the nanoparticle tracking analysis (**Figure 2C**) and transmission electron microscopy (**Figure 2D** and **Figure S4B**) indicated that *S. typhimurium*-released OMV size ranged from 50 to 200 nm. In addition, we confirmed the absence of bacterial contamination in the isolated OMV fractions or the CS fraction (**Figure S4C**) by overnight culture in the LB agar plate to exclude a possibility that free bacteria can affect the results.

Next, *Salmonella*-derived OMVs were treated into BMDMs to examine the innate immune responses against bacterial OMVs. Vybrant Dil-labeled OMVs were clearly detected in the cytosol of BMDMs (**Figure 2E**), suggesting that bacteria-derived OMVs can deliver bacterial components into the cytoplasm of host cells. Of note, treatment with OMVs isolated from *S. typhimurium* (SL1344 or 14028s) caused a robust caspase-1 activation and IL-1β secretion in BMDMs (**Figure 2F**). *S. typhimurium* SL1344 OMV-triggered caspase-1 activation was markedly impaired by flagellin deficiency (**Figure 2G**). Consistently, OMVs from flagellin-deficient *Salmonella* SL1344 induced significantly less IL-1β secretion than wild-type *Salmonella* OMVs in BMDMs (**Figure 2H**). OMVs from flagellin-deficient *S. typhimurium* strain 14028s also failed to trigger robust caspase-1 activation and IL-1β production (**Figures S5A, B**). In contrast, flagellin deficiency did not affect the *Salmonella* OMV-induced IL-6 production (**Figure S5C**).

To examine whether *Salmonella* OMVs did act to transport bacterial proteins to the cytosol, we blocked phagocytosis using cytochalasin D and clathrin-mediated endocytosis using Pitstop 2 (PS2), respectively. PS2 treatment, but not cytochalasin D treatment, markedly inhibited inflammasome activation in BMDMs in response to *S. typhimurium*-released OMVs and CS (**Figure 2I**). PS2 treatment also significantly inhibited cytosolic entry of labeled-OMVs (**Figures 2J, K**), indicating that entry of *S. typhimurium* OMVs was facilitated by clathrin-mediated endocytosis. Consistently, PS2 significantly reduced *Salmonella* OMV-triggered IL-1β secretion (**Figure 2L**). These findings support the hypothesis that *Salmonella* OMVs function as a cargo carrier for cytosolic delivery of bacterial proteins through clathrin-mediated endocytosis.

### *Salmonella typhimurium*-Released Outer Membrane Vesicles Promote NLRC4-Dependent Inflammasome Activation

**Figure 3:**
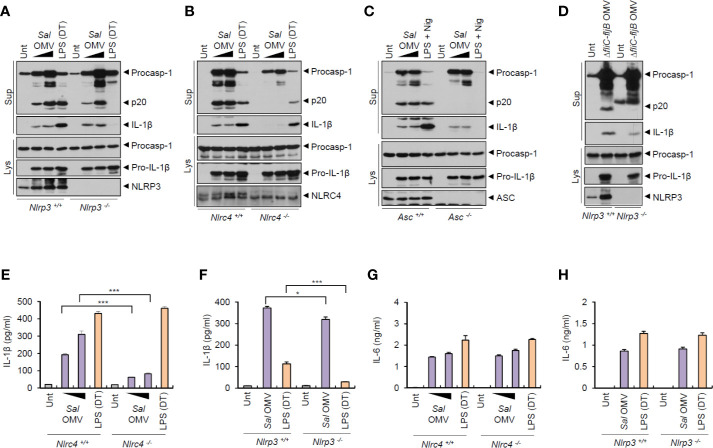
*Salmonella*-released outer membrane vesicles promotes NLRC4-dependent inflammasome activation. **(A, B)** Immunoblots from *Nlrp3* ^+/+^ or *Nlrp3* ^−/−^ **(A)** or *Nlrc4* ^+/+^ or *Nlrc4* ^−/−^ **(B)** mice BMDMs treated with *S. typhimurium* OMVs (3 or 10 µg/ml, 6 h, A; 5 or 12.5 µg/ml, 6 h, B), or treated with Pam3CSK4 (1 µg/ml, 4 h), followed by the transfection of LPS (1 µg/ml, 6 h) using a DOTAP (DT). **(C)** Immunoblots from *Asc* ^+/+^ or *Asc* ^−/−^ immortalized BMDMs treated with *S. typhimurium* OMVs (5 or 12.5 µg/ml, 6 h), or primed with LPS (1 µg/ml, 6 h), followed by the treatment with nigericin (5 µM, 40 min). **(D)** Immunoblots from *Nlrp3* ^+/+^ or *Nlrp3* ^−/−^ mice BMDMs treated with *ΔfliC*–*fljB* *S. typhimurium* OMVs (10 µg/ml) for 6 h. **(E, F)** Quantification of IL-1β in the culture supernatants of *Nlrc4* ^+/+^ or *Nlrc4* ^−/−^ **(E)** or *Nlrp3* ^+/+^ or *Nlrp3* ^−/−^ **(F)** mice BMDMs treated with *S. typhimurium* OMVs (1 or 7 µg/ml, E; 10 µg/ml, F) for 8 h, or treated with Pam3CSK4 (1 µg/ml, 3 h), followed by the transfection of LPS (2 µg/ml, E; 1 µg/ml, F) for 6 h. (*n* = 3) **(G, H)** Quantification of IL-1β in the culture supernatants of *Nlrc4* ^+/+^ or *Nlrc4* ^−/−^ **(G)** or *Nlrp3* ^+/+^ or *Nlrp3* ^−/−^ **(H)** mice BMDMs treated with as same as in **(E, F)**. (*n* = 3) Culture supernatants (Sup) or cellular lysates (Lys) were immunoblotted with the indicated antibodies. Data were expressed as the mean ± SEM. Asterisks indicate significant differences compared with the group in the *Nlrp3* ^+/+^ cells. (*P < 0.05, ***P < 0.001).

To elucidate the mechanisms underlying the inflammasome activation by *S. typhimurium*-derived OMVs, we examined which sensor molecule was associated with OMV-mediated inflammasome activation. Previous studies found that OMVs from *Escherichia coli* trigger caspase-11-mediated non-canonical inflammasome activation *via* LPS delivery into the cytoplasm, leading to NLRP3-dependent IL-1β secretion (18, 19). Unexpectedly, the results indicated that *S. typhimurium* OMVs were capable of inducing caspase-1 activation, even in *Nlrp3*-deficient BMDMs (**Figure 3A**). As a control for caspase-11-mediated non-canonical inflammasome signaling, cytosolic delivery of LPS using DOTAP caused NLRP3-dependent caspase-1 activation (**Figure 3A**). In contrast, *S. typhimurium* OMV-induced caspase-1 activation was remarkably abolished in *Nlrc4*- and *Asc*-deficient BMDMs (**Figures 3B, C**). This result indicate that NLRC4 was a major sensor molecule for the *S. typhimurium* OMVs. Flagellin-deficient *Salmonella*-released OMVs caused very faint caspase-1 activation in macrophages (**Figure 3D**). This attenuated caspase-1 activation by flagellin-lacking OMVs apparently depended on the expression of NLRP3 in BMDMs (**Figure 3D**). Consistently, flagellin-deficient *S. typhimurium* OMVs induced a weak IL-1β production in a NLRP3-dependent manner (**Figure S5D**).

In support of these findings, *S. typhimurium* OMV-induced IL-1β production was significantly impaired in *Nlrc4*-knockout macrophages (**Figure 3E**). It was only very slightly reduced in NLRP3-knockout macrophages (**Figure 3F**). But *Salmonella* OMV treatment resulted in robust NLRC4- and NLRP3-independent IL-6 production (**Figures 3G, H**). Taken together, these findings indicate that *Salmonella*-released OMVs can trigger the activation of NLRC4/ASC-mediated inflammasome signaling pathways in host macrophages.

### Flagellated Bacteria-Released Outer Membrane Vesicles Mediate NLRC4-Dependent Inflammasome Signaling

**Figure 4:**
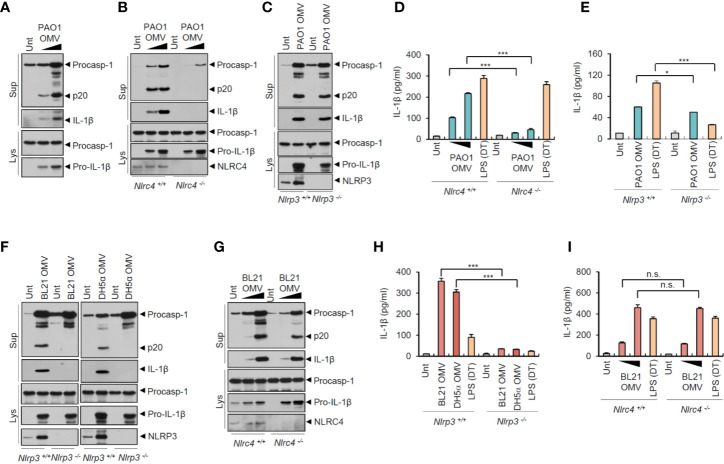
Flagellated bacteria-released outer membrane vesicles trigger NLRC4-dependent inflammasome activation. **(A)** Immunoblots from mouse BMDMs treated with OMV (0.5 or 3 µg/ml) isolated from *Pseudomonas aeruginosa* PAO1 for 8 h. **(B)** Immunoblots from *Nlrc4* ^+/+^ or *Nlrc4* ^−/−^ mice BMDMs treated with *P. aeruginosa* OMV (1 or 7 µg/ml) for 8 h. **(C)** Immunoblots from *Nlrp3* ^+/+^ or *Nlrp3* ^−/−^ mice BMDMs treated with *P. aeruginosa* OMV (5 µg/ml) for 8 h. **(D, E)** Quantification of IL-1β in the culture supernatants of *Nlrc4* ^+/+^ or *Nlrc4* ^−/−^ **(D)** or *Nlrp3* ^+/+^ or *Nlrp3* ^−/−^ **(E)** mice BMDMs treated with *P. aeruginosa* OMV (1 or 7 µg/ml, D; 5 µg/ml, E) for 8 h, or treated with Pam3CSK4 (1 µg/ml, 3 h), followed by the transfection of LPS (2 µg/ml, D; 1 µg/ml, E) for 6 h. (*n* = 3) **(F)** Immunoblots from *Nlrp3* ^+/+^ or *Nlrp3* ^−/−^ mice BMDMs treated with *E. coli* BL21 or DH5α OMVs (5 µg/ml) for 8 h. **(G)** Immunoblots from *Nlrc4* ^+/+^ or *Nlrc4* ^−/−^ mice BMDMs treated with *E. coli* BL21 OMVs (1 or 5 µg/ml) for 8 h. **(H, I)** Quantification of IL-1β in the culture supernatants of *Nlrp3* ^+/+^ or *Nlrp3* ^−/−^ **(H)** or *Nlrc4* ^+/+^ or *Nlrc4* ^−/−^ **(I)** mice BMDMs treated with *E. coli* BL21-derived OMVs (5 µg/ml, H; 1 or 5 µg/ml, I) or DH5*α*-derived OMVs (5 µg/ml, H) for 8 h, or treated with Pam3CSK4 (1 µg/ml, 3 h), followed by the transfection of LPS (1 µg/ml, H; 2 µg/ml, I) for 6 h. (*n* = 3) Culture supernatants (Sup) or cellular lysates (Lys) were immunoblotted with the indicated antibodies. Data were expressed as the mean ± SEM. Asterisks indicate significant differences compared with the group in the *Nlrp3* ^+/+^ cells. (*P < 0.05, ***P < 0.001, n.s. not significant).

To examine whether OMVs derived from bacteria other than *Salmonella* can induce NLRC4-mediated inflammasome activation, we tested the effects of the other Gram-negative bacterial pathogen, *Pseudomonas aeruginosa* PAO1. Consistent with the *S. typhimurium* results, *P. aeruginosa*-released OMVs also caused a robust caspase-1 activation and active IL-1β secretion (**Figure 4A**). This *P. aeruginosa* OMV-driven inflammasome activation depended on NLRC4, but not on NLRP3 (**Figures 4B, C**). Consistent with these findings, *P. aeruginosa* OMV treatment resulted in robust secretion of IL-1β in a NLRC4-dependent manner (**Figure 4D**). Instead, NLRP3 deficiency caused a slight reduction in IL-1β production by *Pseudomonas* OMVs (**Figure 4E**).

Given that flagellin may be the main stimulus for *Salmonella*-released OMV-induced NLRC4 inflammasome activation, we examined the roles of OMVs from non-flagellated bacteria, such as *E. coli* strains BL21 and DH5*α*. Unlike *Salmonella* or *Pseudomonas* OMVs, *E. coli* (BL21 and DH5*α*)-released OMVs induced robust caspase-1 activation only in NLRP3-expressing macrophages (**Figure 4F**). In contrast, NLRC4 deficiency did not affect *E. coli* OMV-driven caspase-1 activation (**Figure 4G**). Consistent with these results, non-flagellated *E. coli* OMVs promoted NLRP3-dependent, but NLRC4-independent, IL-1β secretion (**Figures 4H, I**). Furthermore, *E. coli* OMV-induced inflammasome activation was blocked by NLRP3-selective inhibitor MCC950 (**Figure S6**). These findings indicate that non-flagellated bacteria-released OMVs induce NLRP3 inflammasome activation probably *via* triggering caspase-11-mediated non-canonical inflammasome signaling. In contrast, flagellated bacteria-released OMVs can facilitate canonical NLRC4 inflammasome pathways.

### *Salmonella typhimurium* Outer Membrane Vesicles-Contained Flagellin Triggers Inflammasome Activation in a GBP2-Independent Manner

**Figure 5:**
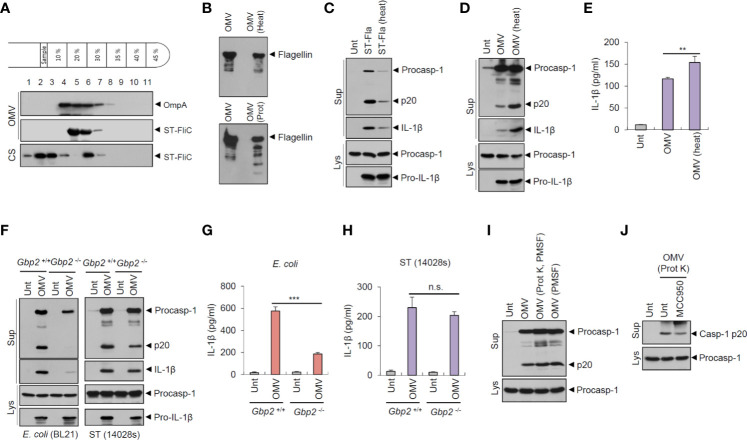
*Salmonella*-released outer membrane vesicles exhibit heat-resistant and GBP2-independent inflammasome activation potential. **(A)** Immunoblots of OptiPrep density gradient fractions of *S. typhimurium* OMVs. **(B)** Immunoblots of the extracts of OMVs in the presence of heat treatment (97°C, 30 min) or proteinase K treatment (10 µg/ml, 30 min) by anti-ST-flagellin antibody. **(C)** Immunoblots from mouse BMDMs primed with LPS (0.25 µg/ml, 3 h), followed by the treatment with intact or heat-treated *S. typhimurium* flagellin (250 ng/ml) for 6 h. **(D)** Immunoblots of mouse BMDMs treated with intact or heat-treated *S. typhimurium* OMVs (5 µg/ml) for 6 h. **(E)** Quantification of IL-1β in the culture supernatants of mouse BMDMs treated with intact or heat-treated *S. typhimurium* OMVs for 6 h. (*n* = 5). **(F)** Immunoblots from *Gbp2* ^+/+^ or *Gbp2* ^−/−^ mice BMDMs treated with *E. coli* BL21 OMVs (5 µg/ml) or *S. typhimurium* (14028s) OMVs (5 µg/ml) or for 8 h. **(G, H)** Quantification of IL-1β in the culture supernatants of *Gbp2* ^+/+^ or *Gbp2* ^−/−^ mice BMDMs treated as same as **(F)** (*n* = 3). **(I, J)** Immunoblots of mouse BMDMs treated with intact or proteinase-treated *S. typhimurium* OMVs (10 µg/ml) in the presence of MCC 950 (100 nM) as indicated for 8 h. After proteinase treatment, PMSF was added to eliminate proteinase K activity. Culture supernatants (Sup) or cellular lysates (Lys) were immunoblotted with the indicated antibodies. Data were expressed as the mean ± SEM. Asterisks indicate significant differences. (**P < 0.01, ***P < 0.001, n.s. not significant).

As the results indicated, flagellin in the OMVs had a pivotal role in *Salmonella* OMV-induced caspase-1 activation. We thus examined whether flagellin was indeed located in the OMVs derived from *Salmonella* culture. Isolated OMVs were further fractionated using an OptiPrep density gradient medium. Flagellin was detected in the same fraction as in OmpA, an unique OMV marker (**Figure 5A**), indicating that flagellin was present in the *S. typhimurium*-released OMVs. Moreover, flagellin was observed in the low density fraction of bacterial CS, but not of OMVs (**Figure 5A**), suggesting that isolated OMVs contain no or very little free flagellin outside OMVs. Nevertheless, to further confirm whether flagellin is present as inserted forms in the OMV membrane or inside OMVs, isolated OMVs were subjected to heat and proteinase K treatment. Flagellin in the OMVs was almost intact against heat- and proteinase treatment (**Figure 5B**), while flagellin in the bacterial CS was completely degraded by proteinase treatment (**Figure S1B**). Indeed, heat treatment significantly impaired bacterial CS-induced inflammasome activation (**Figures 1F, G**). Similarly, heat-inactivation of recombinant *Salmonella* flagellin protein greatly impaired inflammasome activation (**Figure 5C**). However, heat treatment did not inhibit rather increase *S. typhimurium* OMV-triggered caspase-1 activation and IL-1β secretion (**Figures 5D, E**). These results demonstrate that OMVs may function as a cargo for cytosolic delivery of flagellin against heat- or proteinase-induced damages.

Guanylate-binding proteins (GBPs) are required for bacterial OMV-mediated non-canonical inflammasome activation in the previous studies (19, 24). The results suggest that GBP2 and GBP5 facilitate the release of LPS from OMVs, which enables the interaction between LPS and caspase-11. In this context, we investigated the potential involvement of GBP2 on *S. typhimurium* OMV-induced inflammasome activation. GBP2 deficiency clearly abrogated caspase-1 activation and IL-1β secretion by OMVs isolated from *E. coli* culture (**Figures 5F, G**). In contrast to *E. coli*-derived OMVs, wild-type *Salmonella*-derived OMVs caused similar levels of caspase-1 processing and IL-1β production from *Gbp2*-deficient macrophages (**Figures 5F, H**). However, flagellin-deficient (Δ*fliC*–*fljB*) *S. typhimurium* OMVs led to a weak GBP2-dependent IL-1β production (**Figure S7**), indicating that *S. typhimurium* OMVs are also able to mediate non-canonical inflammasome activation to a lesser degree. These results demonstrate that *E. coli* OMVs and flagellin-deficient *S. typhimurium* OMVs can trigger GBP2-NLRP3-mediated non-canonical inflammasome activation.

In addition, we further examined inflammasome-activating capacity of proteinase-treated *S. typhimurium* OMVs to exclude a possibility that free flagellin contamination in the isolated OMVs may affect our results. Proteinase-treated OMVs caused a similar extent of caspase-1 activation to intact OMVs (**Figure 5I**). Furthermore, proteinase-treated OMVs induced a robust caspase-1 activation regardless of MCC950 treatment (**Figure 5J**). These observations further support that *S. typhimurium* OMVs can trigger NLRC4 inflammasome signaling *via* OMV-associated flagellin.

### *Salmonella typhimurium* Outer Membrane Vesicles Drive More Rapid NLRC4-Dependent Inflammasome Activation in a Flagellin-Dependent Manner Than *E. coli*-Derived Outer Membrane Vesicles

**Figure 6:**
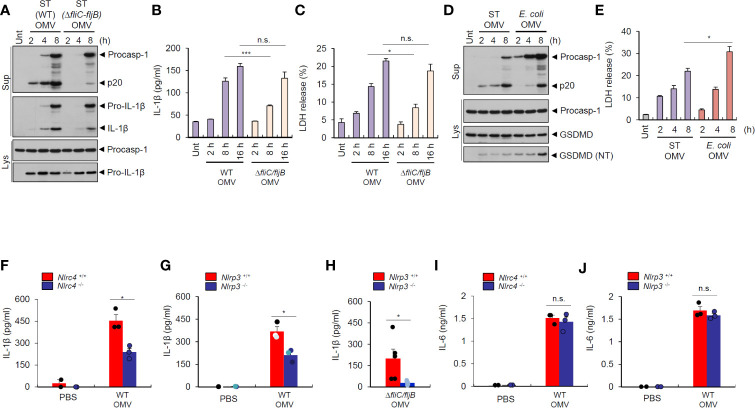
*Salmonella*-released outer membrane vesicles trigger *in vivo* NLRC4-dependent interleukin-1β secretion. **(A)** Immunoblots from mouse BMDMs treated with wild-type (WT) or *ΔfliC*–*fljB S. typhimurium* (SL1344) OMV (5 µg/ml) for 2, 4, 8 h. **(B)** Quantification of IL-1β in the culture supernatants of mouse BMDMs treated with WT or *ΔfliC*–*fljB S. typhimurium* (SL1344) OMVs (5 µg/ml) for 2, 8, 16 h. (*n* = 3) **(C)** Quantification of LDH release into culture supernatants of mouse BMDMs treated with WT or *ΔfliC*–*fljB S. typhimurium* (SL1344) OMVs (5 µg/ml) for 2, 8, 16 h. (*n* = 3) **(D)** Immunoblots from mouse BMDMs treated with *S. typhimurium* (SL1344) or *E. coli* (BL21) OMVs (5 µg/ml) for 2, 4, 8 h as indicated. GSDMD, gasdermin D **(E)** Quantification of LDH release into culture supernatants of mouse BMDMs treated with *S. typhimurium* (SL1344) or *E. coli* (BL21) OMVs (5 µg/ml) for 2, 4, 8 h as indicated. (*n* = 3). **(F, G)** Quantification of IL-1β in the peritoneal lavage fluid of *Nlrc4* ^+/+^ or *Nlrc4* ^−/−^ **(F)**, or *Nlrp3* ^+/+^ or *Nlrp3* ^−/−^ **(G)** mice 6 h after intraperitoneal injection of PBS or *S. typhimurium* OMVs (50 µg/mice). (*n* = 2, PBS; *n* = 3, OMV) **(H)** Quantification of IL-1β in the peritoneal lavage fluid of *Nlrp3* ^+/+^ or *Nlrp3* ^−/−^ mice 6 h after intraperitoneal injection of *ΔfliC*–*fljB* *S. typhimurium* OMVs (50 µg/mice). (*n* = 5, *Nlrp3* ^+/+^; *n* = 6, *Nlrp3* ^−/−^) **(I, J)** Quantification of IL-6 in the peritoneal lavage fluid of *Nlrc4* ^+/+^ or *Nlrc4* ^−/−^ **(I)**, or *Nlrp3* ^+/+^ or *Nlrp3* ^−/−^ **(J)** mice 6 h after intraperitoneal injection of PBS or *S. typhimurium* OMVs (50 µg/mice). (*n* = 2, PBS; *n* = 3, OMV) Culture supernatants (Sup) or cellular lysates (Lys) were immunoblotted with the indicated antibodies. Data were expressed as the mean ± SEM. Asterisks indicate significant differences. (*P < 0.05, ***P < 0.001, n.s. not significant).

To further evaluate the role of flagellin for inflammasome-stimulating potential of *Salmonella* OMVs, we examined time-dependent inflammasome activity induced by wild-type and flagellin-deficient *S. typhimurium* OMVs. We found that flagellin-deficient *Salmonella*-released OMVs caused a delayed and weaker inflammasome activation than wild-type *S. typhimurium* OMVs in BMDMs (**Figures 6A, B**). Interestingly, flagellin-deficient *Salmonella* OMVs induced a significant weaker cell death of macrophages, as measured by LDH release, until 8 h post OMV treatment compared to wild-type *Salmonella* OMVs (**Figure 6C**). However, prolonged (16 h) treatment with *Salmonella* OMVs caused similar IL-1β production and pyroptotic cell death irrespective of flagellin expression (**Figures 6B, C**).

Then, we checked time-dependent inflammasome response from macrophages upon treatment with *Salmonella*- or *E.coli*-derived OMVs. *S. typhimurium* OMVs promoted rapid caspase-1 activation as early as 2 h post treatment, whereas *E. coli* OMVs did not induce robust caspase-1 activation until 8 h-treatment (**Figure 6D**). These findings suggest that *S. typhimurium* OMV-associated flagellin can trigger more rapid host inflammasome activation than *E. coli* OMV-bound LPS. Instead, *E. coli* OMVs promoted stronger gasdermin D cleavage (**Figure 6D**), as determined by the presence of cleaved GSDMD (NT) in the cytosol, and pyroptotic cell death (**Figure 6E**) than *S. typhimurium* OMVs at 8 h-treatment to macrophages. These results propose that NLRC4 inflammasome machinery is a critical sensor for rapid detection of flagellated bacteria-released OMVs as a host defense mechanism, whereas non-flagellated bacteria-induced caspase-11 non-canonical inflammasome signaling rather contributes to pyroptosis in response to bacterial OMV-delivered LPS.

To determine *in vivo* role of NLRC4 against *S. typhimurium*–released OMVs, we intraperitoneally injected bacterial OMVs into mice. Analysis of peritoneal lavage contents from mice revealed that intraperitoneal challenge with *S. typhimurium*-released OMVs caused robust IL-1β production (**Figure 6F**). *Nlrc4*-deficient mice showed significantly reduced IL-1β production (**Figure 6F**) after *S. typhimurium* OMV challenge. Interestingly, NLRP3 deficiency significantly reduced IL-1β production in the peritoneal lavage of mice after intraperitoneal injection of *Salmonella*-derived OMVs (**Figure 6G**). Flagellin-deficient *S. typhimurium* OMVs also caused NLRP3-dependent IL-1β production from mice (**Figure 6H**), indicating that *Salmonella*-derived OMVs can facilitate both NLRC4- and NLRP3-mediated inflammasome activation in mice. However, IL-6 production was promoted regardless of NLRC4 and NLRP3 presence in the peritoneal lavage of mice upon *S. typhimurium* OMV challenge (**Figures 6I, J**). These findings further support the hypothesis that *Salmonella*-derived OMVs can induce *in vivo* NLRC4-mediated canonical inflammasome activation in addition to caspase-11 non-canonical inflammasome activation.

## Discussion

Gram-negative bacteria-released OMVs can facilitate the spread of bacterial pathogenicity through delivery of virulence factors and antibiotic resistance genes (16). Bacterial OMVs can also trigger host innate sensing machinery (*e.g.*, inflammasome pathways), which helps to increase bacterial clearance (17). In particular, bacteria-released OMVs promote caspase-11-mediated non-canonical inflammasome responses *via* OMV-bound LPS (18, 24, 25). OMV-mediated intracellular delivery of LPS results in the activation of caspase-11, a cytosolic LPS sensor, with the help of GBPs (19). Active caspase-11 then induces gasdermin D pore formation in the plasma membrane, which leads to potassium efflux and subsequent NLRP3 inflammasome activation (26). In this context, bacterial OMVs can induce IL-1β secretion in a caspase-11-mediated indirect manner, as described above. However, our study is the first to find that bacterial OMVs can directly trigger host NLRC4-mediated canonical inflammasome activation by delivering bacterial flagellin into the cytoplasm of host cells.

Our results indicate that inflammasome activation by OMVs from flagellated bacteria such as *S. typhimurium* and *P. aeruginosa* was markedly abolished in *Nlrc4*-deficient macrophages, but slightly decreased in *Nlrp3*-deficent cells. Consistently, OMVs released from *S. typhimurium* lacking flagellin and T3SS protein prgJ failed to induce strong inflammasome activation. These findings further support the potential role of bacterial OMV-associated flagellin for the NLRC4 inflammasome activation. Bacterial flagellin is essential for bacterial motility and invasion, and thus is considered a significant virulence factor. Additionally, flagellin is the most abundant protein in the secreted fraction of *S. typhimurium* cultures (27). Previous studies initially suggested that *Salmonella* flagellin is delivered to the cytoplasm of host cells through T3SS-dependent direct secretion and bacterial phagocytosis (22, 28). However, recent studies found that flagellin is also detected in OMVs from *S. typhimurium* and *P. aeruginosa* (29–31). In this context, OMV cargo is capable of delivering bacterial flagellin into the cytoplasm leading to intracellular NLRC4 inflammasome responses.

Our data demonstrate that heat treatment significantly reduced inflammasome-stimulating potential of *Salmonella* CS, but not of *Salmonella* OMVs. Considering that heat treatment did not induce flagellin degradation in the bacterial CS (**Figure S1B**), we speculate that heat treatment may disrupt the conformation of flagellin or prgJ, resulting in the attenuation of the ability to activate the inflammasomes. In contrast, heat treatment slightly increased *Salmonella* OMV-driven inflammasome activation. It is possible that heat may cause a weak disruption of OMV structure, facilitating more efficient delivery to the cytoplasm. These findings suggest that OMVs can protect flagellin from heat-induced damage during the delivery to the target cells.

Somewhat different from the *in vitro* results in macrophages, *in vivo* IL-1β production by *S. typhimurium* OMV was dependent on both NLRC4 and NLRP3 after intraperitoneal challenge with OMVs (**Figure 6**). These *in vivo* results suggest that *Salmonella* OMV-bound LPS was also important for inflammasome activation in the peritoneal lavage contents of mice. Both NLRC4 and NLRP3 are likely to be redundant for detecting *Salmonella*-derived OMVs to trigger inflammasome responses, at least in peritoneal lavage contents. Flagellin was the main stimulant for *S. typhimurium* OMV-driven inflammasome activation in the BMDMs, but flagellin-deficient *S. typhimurium* OMVs also triggered a slight inflammasome activation in a NLRP3-dependent manner (**Figure 3D**). However, we found that flagellin-deficient *S. typhimurium* OMVs caused a delayed inflammasome response in BMDMs compared with wild-type *S. typhimurium* OMVs (**Figure 6A**). Furthermore, flagellated *S. typhimurium* OMVs induced more rapid inflammasome activation than non-flagellated *E. coli* OMVs in BMDMs (**Figure 6D**). These results indicate that at least in macrophages, NLRC4 inflammasome machinery is a more rapid sensor for detecting *Salmonella*-released OMVs than caspase-11. Particularly, NLRC4-mediated pathogen sensing is crucial for host defense against enteric infection, but not against intraperitoneal infection (10). In this context, NLRC4 inflammasome signaling for flagellated bacteria-released OMVs might have a major role in host defense, especially in the gut.

GBPs, interferon-inducible GTPases, are required for clearance of intracellular pathogens by inducing lysis of pathogen-containing vacuoles (32–34). GBPs also facilitates the intracellular interaction of OMV-bound LPS with caspase-11 by exposing lipid A (19). Thus, GBPs have a pivotal role in non-canonical inflammasome activation by OMVs. However, GBP2 seemed not to be critical for inflammasome activation by *S. typhimurium*-released OMVs (**Figure 5H**). These results indicated that GBP2 was not involved in activation of NLRC4 inflammasome signaling by OMV-associated flagellin. The precise role of other GBPs for OMV-mediated NLRC4 inflammasome requires further investigation.

**Figure 7:**
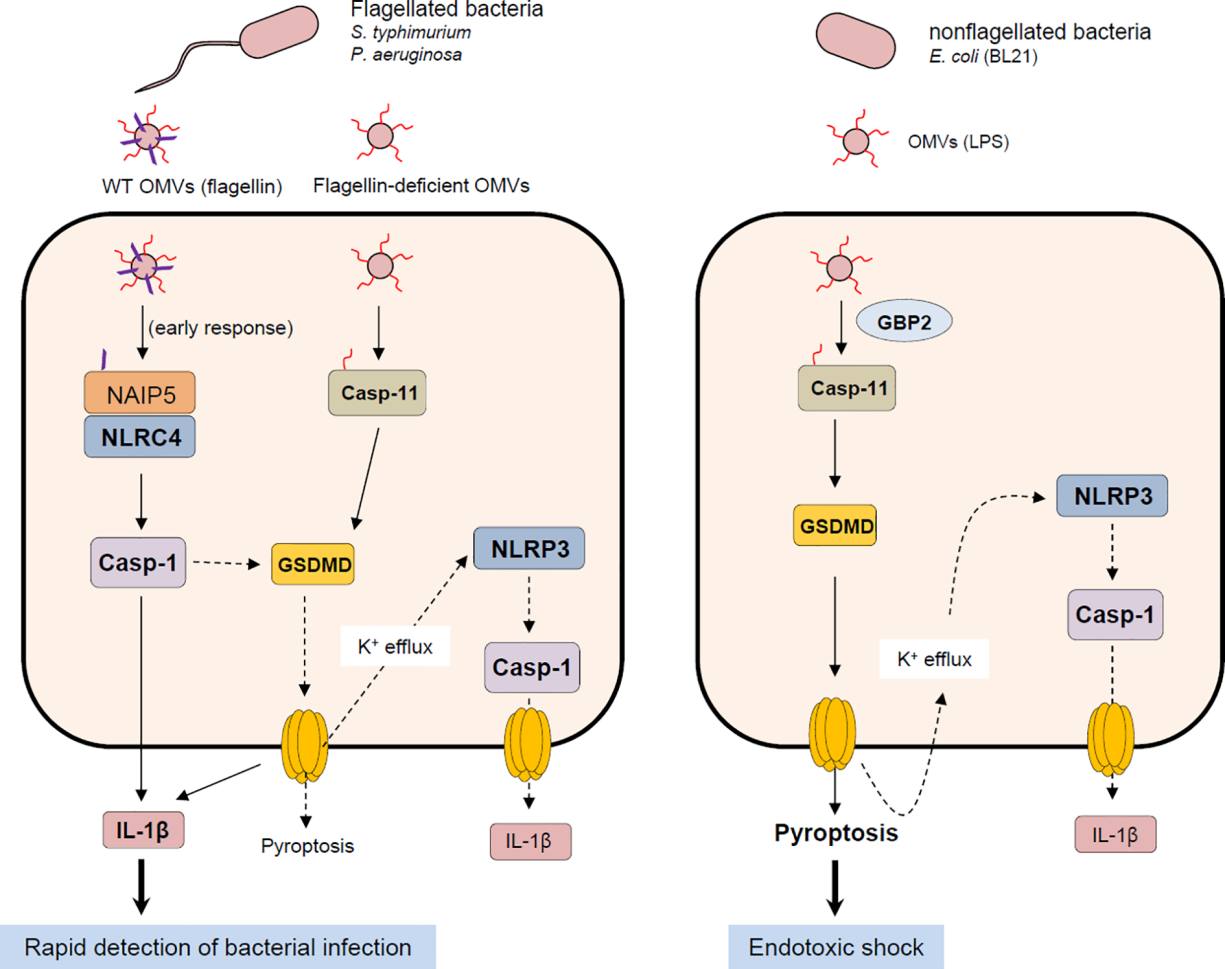
Proposed mechanism of host inflammasome activation by flagellated or non-flagellated bacteria-released outer membrane vesicles. Flagellated bacteria such as *S. typhimurium* or *P. aeruginosa* facilitate the delivery of bacterial flagellin and LPS into the cytoplasm of host cells *via* OMV cargo. In the initial phase of OMV exposure, OMV-delivered flagellin causes the assembly of NLRC4 inflammasome leading to caspase-1 activation and the subsequent IL-1β secretion. Besides flagellin, intracellular OMV-associated LPS can activate caspase-11 non-canonical inflammasome signaling, which includes GSDMD-dependent pyroptosis and NLRP3-mediated caspase-1 activation and IL-1β secretion. On the contrary, OMVs derived from non-flagellated bacteria such as *E. coli* mediates the cytosolic delivery of bacterial LPS. Non-flagellated bacterial -delivered LPS mainly causes the activation of caspase-11 and GSDMD-mediated pyroptosis, contributing to potential endotoxic shock. NAIP5, NLR family, apoptosis inhibitory protein 5; NLRC4, NLR family, CARD domain-containing protein 4; NLRP3, NLR family, pyrin domain-containing protein 3; GSDMD, gasdermin D.

A physiological significance of NLRC4 inflammasome activation by bacterial OMVs remains to be further determined. Based on previous studies and our present results, both caspase-11 non-canonical and NLRC4 canonical inflammasomes promote caspase-1-dependent IL-1β production in response to bacteria-released OMVs (**Figure 7**). Interestingly, both *caspase-11*-deficient and *Gbp*-deficient mice showed increased survival after poly I:C and the subsequent *E. coli* OMV injection (19). This finding indicates that caspase-11 non-canonical inflammasome activation by *E. coli* OMV-associated LPS significantly contributes to endotoxic shock *via* inducing a robust pyroptosis. Instead, NLRC4 inflammasome signaling by flagellated bacteria-released OMVs can contribute to rapid detection of bacterial infection leading to the inhibition of bacterial dissemination and virulence as a host defense mechanism (**Figure 7**). Taken together, our findings suggest that NLRC4 is a bona-fide sensor for detecting flagellated bacteria-released OMVs to mediate inflammasome signaling. These results provide molecular insights into host defense mechanisms against flagellin-containing bacterial pathogens such as *S. typhimurium* or *P. aeruginoasa*.

## Data Availability Statement

The raw data supporting the conclusions of this article will be made available by the authors, without undue reservation.

## Ethics Statement

The animal study was reviewed and approved by Institutional Ethical Committee, Yonsei University College of Medicine.

## Author Contributions

JY designed and conducted most of the experiments in this study. IH and EL conducted the experiments. SS, E-JL, and JR provided critical materials and technical advice. J-WY supervised the entire project and wrote the manuscript with JY. All authors contributed to the article and approved the submitted version.

## Funding

This work was supported by the National Research Foundation of Korea Grant funded by the Korean Government (2015M3A9B6073856, 2017R1A2B2007467, 2020R1A2B5B02001823).

## Conflict of Interest

The authors declare that the research was conducted in the absence of any commercial or financial relationships that could be construed as a potential conflict of interest.
