# Supplementary material for: Bacterial Outer Membrane Vesicle-Mediated Cytosolic Delivery of Flagellin Triggers Host NLRC4 Canonical Inflammasome Signaling
Source: Front Immunol. 2020 Nov 18;11:581165. doi: 10.3389/fimmu.2020.581165 (PMC7708323; doi:10.3389/fimmu.2020.581165)
Supplement: Supplementary file 1 [file DataSheet_1.pdf]

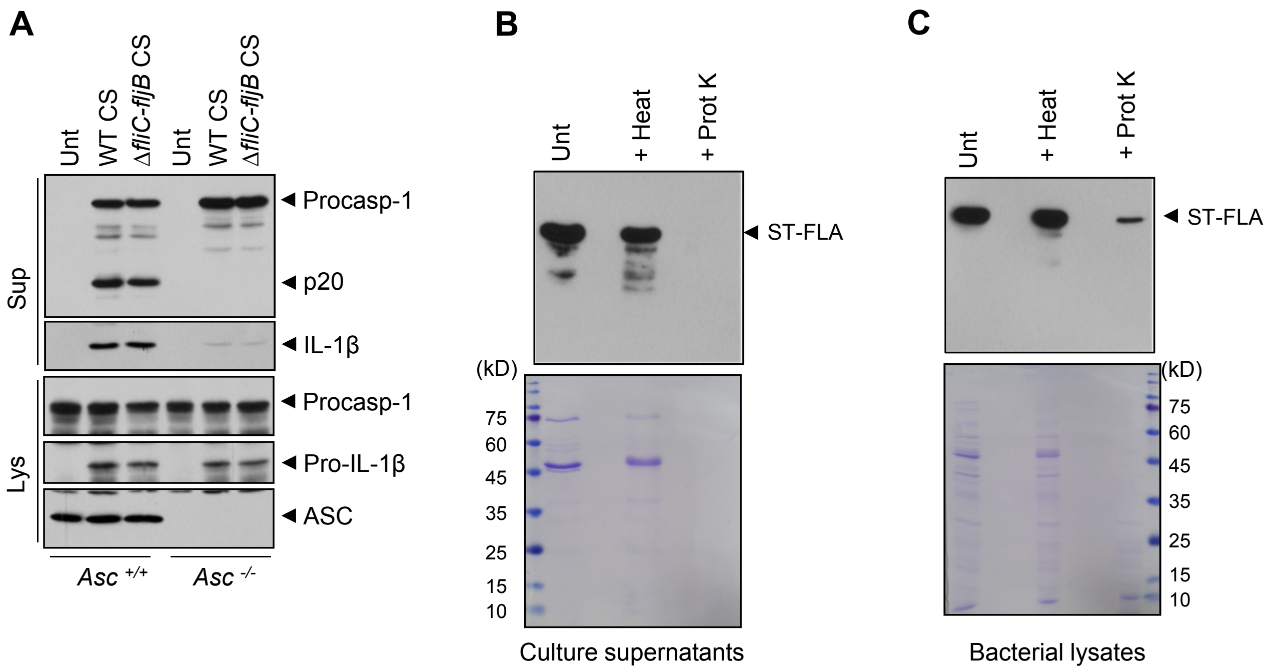


**Supplementary Figure S1. Culture supernatants from *S. typhimurium* promotes ASC-dependent inflammasome activation.** (A) Immunoblots from *Asc* $^{+/+}$ or *Asc* $^{-/-}$ immortalized BMDMs treated with WT or *Δflic-fljB S. typhimurium* CS (1/10 vol of medium) for 6 h. Culture supernatants (Sup) or cellular lysates (Lys) were immunoblotted with the indicated antibodies. Unt, untreated. (B, C) Immunoblots (upper panel) by anti-ST-FliC antibody and coomassie stained gels (lower panel) of *S. typhimurium* culture supernatants (B) or lysates (C) after heat treatment (97°C, 30 min) or proteinase K treatment (10 μg/ml, 30 min).

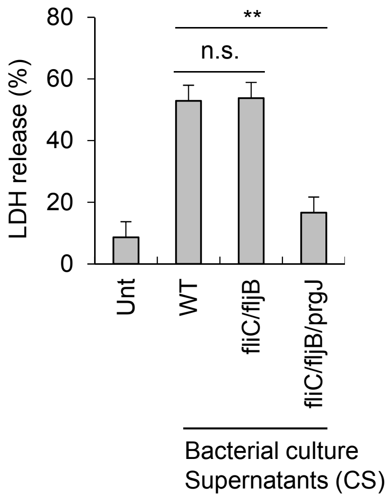


**Supplementary Figure S2. Culture supernatants from *S. typhimurium* promotes macrophage cell death.** Quantification of LDH release into culture supernatants of mouse BMDMs treated with WT or *Δflic-fljB* or *Δflic-fljB-prgJ S. typhimurium* (SL1344) CS (1/20 vol of medium) for 6 h. ($n$ = 3) Unt, untreated. Asterisk indicates significant difference. (**$P$<0.005, n.s. not significant, one-way ANOVA)

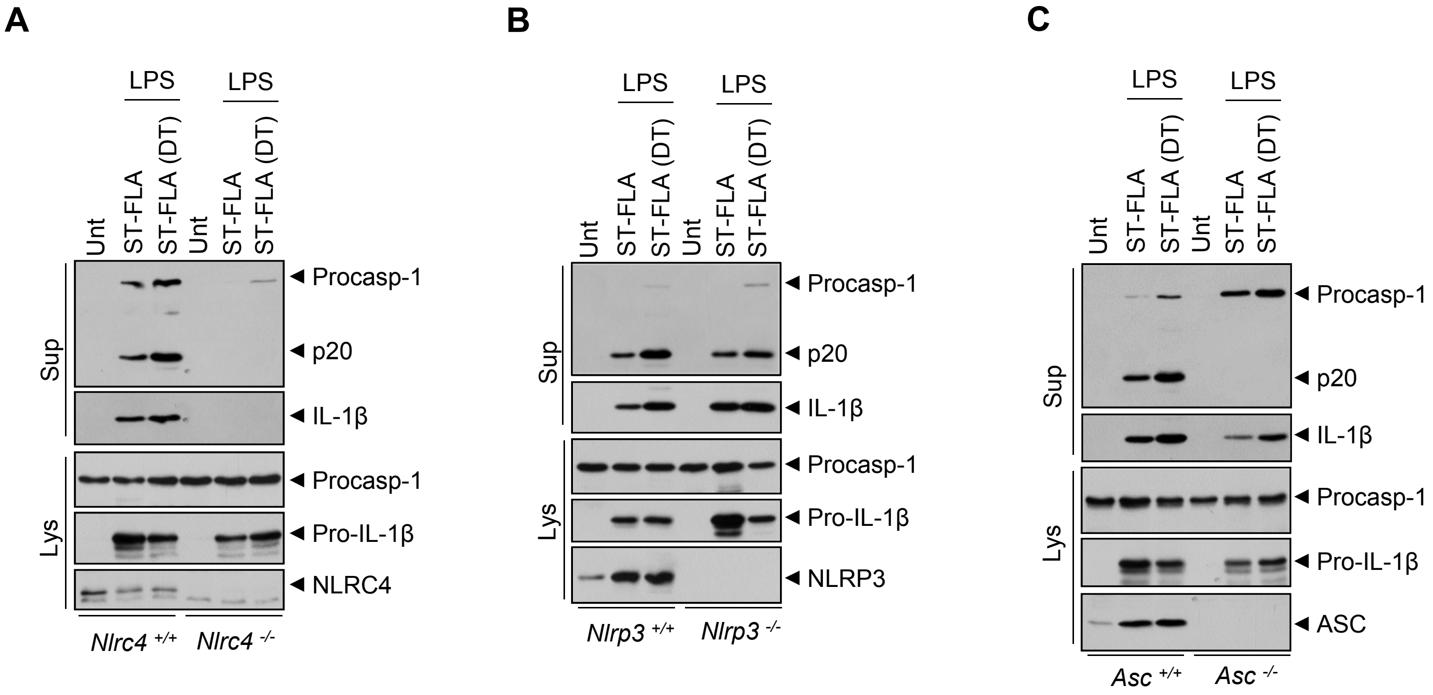


**Supplementary Figure S3. Recombinant flagellin from *S. typhimurium* induces NLRC4 inflammasome activation.** (**A, B**) Immunoblots from *Nlrc4* $^{+/+}$ or *Nlrc4* $^{-/-}$ (A) and *Nlrp3* $^{+/+}$ or *Nlrp3* $^{-/-}$ (B) mice BMDMs treated with LPS (0.25 µg/ml, 3 h), followed by the treatment with *S. typhimurium* flagellin (250 ng/ml, 6 h) with or without DOTAP (DT) premixing. (**C**) Immunoblots from *Asc* $^{+/+}$ or *Asc* $^{-/-}$ immortalized BMDMs treated with LPS (0.25 µg/ml, 3 h), followed by the treatment with *S. typhimurium* flagellin (250 ng/ml, 6 h) with or without DT premixing. Culture supernatants (Sup) or cellular lysates (Lys) were immunoblotted with the indicated antibodies. Unt, untreated.

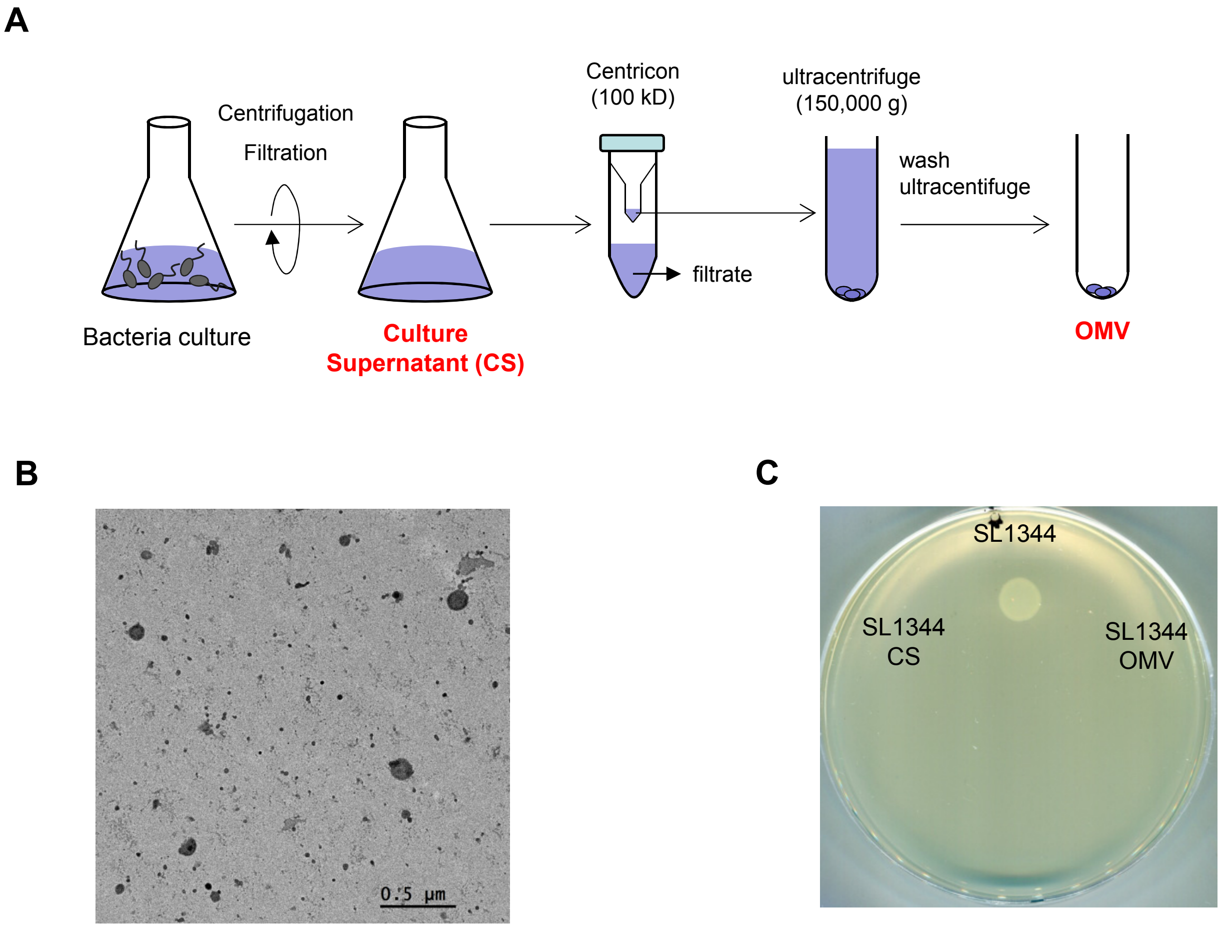


**Supplementary Figure S4. Isolation of *S. typhimurium*-released OMVs. (A)** Illustration of experimental scheme to isolate bacterial outer membrane vesicles (OMVs). (**B**) Transmission electron microscopy of OMVs isolated from wild-type *S. typhimurium*. Scale bar, 0.5 μm. (**C**) LB agar plate inoculated with *S. typhimurium* SL1344, SL1344 CS and SL1344 OMV.

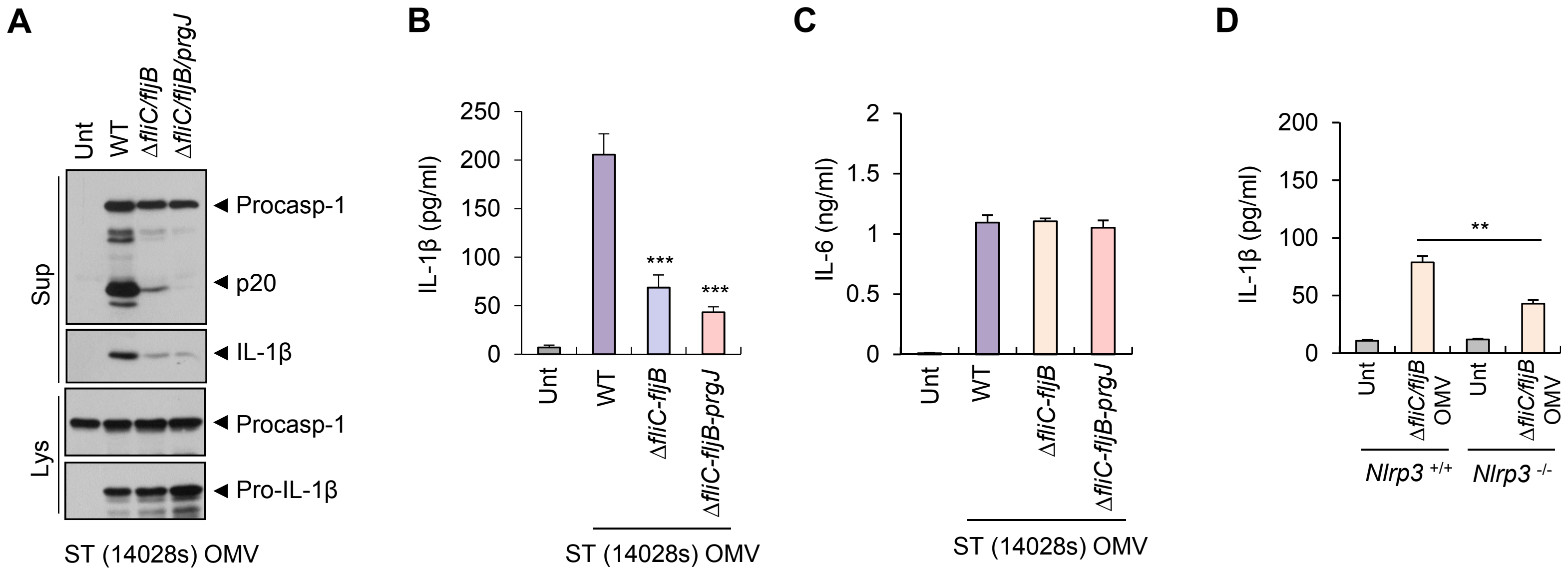


**Supplementary Figure S5. OMV-associated flagellin is critical for the inflammasome activation by *S. typhimurium*-released OMVs. (A)** Immunoblots from mouse BMDMs treated with WT, *Δflic-fljB*, or *Δflic-fljB-prgJ S. typhimurium* (14028s) OMVs (5 μg/ml, 8 h). Culture supernatants (Sup) or cellular lysates (Lys) were immunoblotted with the indicated antibodies. (**B**) Quantification of IL-1β in the culture supernatants of mouse BMDMs treated with WT, *Δflic-fljB*, or *Δflic-fljB-prgJ S. typhimurium* (14028s) OMVs (5 μg/ml, 8 h). (**C)** Quantification of IL-6 in the culture supernatants of mouse BMDMs treated with WT, *Δflic-fljB*, or *Δflic-fljB-prgJ S. typhimurium* (14028s) OMVs (5 μg/ml, 8 h). (**D)** Quantification of IL-1β in the culture supernatants of *Nlrp3* $^{+/+}$ or *Nlrp3* $^{-/-}$ mice BMDMs treated with *Δflic-fljB S. typhimurium* (SL1344) OMV (5 μg/ml, 8 h) ($n$ = 5). Asterisks indicate significant difference. (**$P$<0.005, ***$P$<0.001)

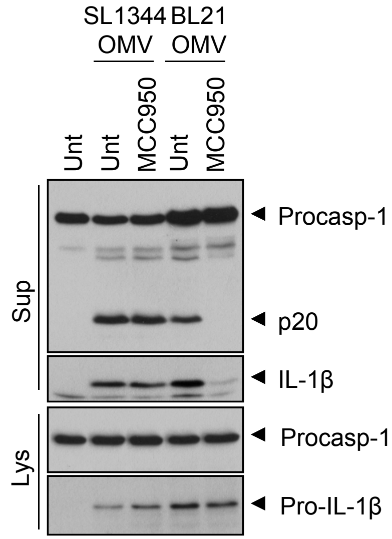


**Supplementary Figure S6. *E. coli*-derived OMVs caused a NLRP3-dependent caspase-1 processing.** Immunoblots from mouse BMDMs treated with *S. typhimurium* (SL1344) OMVs (5 μg/ml, 8 h) or *E. coli* (BL21) OMVs (5 μg/ml, 8 h) in the presence of MCC950 (100 nM). Culture supernatants (Sup) or cellular lysates (Lys) were immunoblotted with the indicated antibodies.

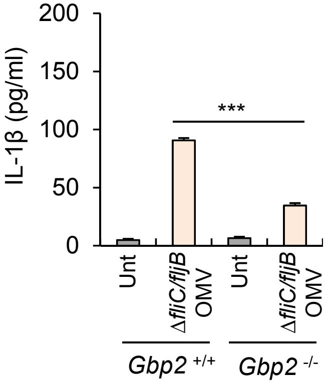


**Supplementary Figure S7. Flagellin-deficient *S. typhimurium*-derived OMVs caused NLRP3-dependent IL-1β production.** Quantification of IL-1β in the culture supernatants of *Gbp2* $^{+/+}$ or *Gbp2* $^{-/-}$ mice BMDMs treated with *Δflic-fljB S. typhimurium* (SL1344) OMV (5 μg/ml, 8 h). (*n* = 5). Asterisk indicates significant difference. (****P*<0.001)
